# RNA polymerase inaccuracy underlies SARS-CoV-2 variants and vaccine heterogeneity

**DOI:** 10.21203/rs.3.rs-1690086/v1

**Published:** 2022-06-02

**Authors:** Catherine C. Bradley, Alasdair J.E. Gordon, Chen Wang, Matthew B. Cooke, Brendan F. Kohrn, Scott R. Kennedy, Olivier Lichtarge, Shannon E. Ronca, Christophe Herman

**Affiliations:** 1 Department of Molecular and Human Genetics, Baylor College of Medicine; Houston, Texas 77030, USA; 2 Baylor College of Medicine Medical Scientist Training Program; Houston, Texas 77030, USA; 3 Robert and Janice McNair Foundation/ McNair Medical Institute M.D./Ph.D. Scholars program; Houston, Texas 77030, USA; 4 Department of Laboratory Medicine and Pathology, University of Washington; Seattle, WA 98195, USA; 5 Feigin Biosafety Level 3 Facility, Texas Children’s Hospital; Houston, Texas 77030, USA; 6 National School of Tropical Medicine, Department of Pediatrics Tropical Medicine, Texas Children’s Hospital and Baylor College of Medicine; Houston, Texas 77030, USA; 7 Department of Molecular Virology and Microbiology, Baylor College of Medicine; Houston, Texas 77030, USA; 8 Dan L. Duncan Cancer Center, Baylor College of Medicine; Houston, TX 77030, USA

## Abstract

Both the SARS-CoV-2 virus and its mRNA vaccines depend on RNA polymerases (RNAP)^1,2^; however, these enzymes are inherently error-prone and can introduce variants into the RNA^3^. To understand SARS-CoV-2 evolution and vaccine efficacy, it is critical to identify the extent and distribution of errors introduced by the RNAPs involved in each process. Current methods lack the sensitivity and specificity to measure *de novo* RNA variants in low input samples like viral isolates^3^. Here, we determine the frequency and nature of RNA errors in both SARS-CoV-2 and its vaccine using a targeted Accurate RNA Consensus sequencing method (tARC-seq). We found that the viral RNA-dependent RNAP (RdRp) makes ~1 error every 10,000 nucleotides – higher than previous estimates^4^. We also observed that RNA variants are not randomly distributed across the genome but are associated with certain genomic features and genes, such as S (Spike). tARC-seq captured a number of large insertions, deletions and complex mutations that can be modeled through non-programmed RdRp template switching. This template switching feature of RdRp explains many key genetic changes observed during the evolution of different lineages worldwide, including Omicron. Further sequencing of the Pfizer-BioNTech COVID-19 vaccine revealed an RNA variant frequency of ~1 in 5,000, meaning most of the vaccine transcripts produced *in vitro* by T7 phage RNAP harbor a variant. These results demonstrate the extraordinary genetic diversity of viral populations and the heterogeneous nature of an mRNA vaccine fueled by RNAP inaccuracy. Along with functional studies and pandemic data, tARC-seq variant spectra can inform models to predict how SARS-CoV-2 may evolve. Finally, our results may help improve future vaccine development and study design as mRNA therapies continue to gain traction.

Since the COVID-19 pandemic began, we have witnessed the repeated emergence of new SARS-CoV-2 lineages and viral variants of concern (VOC) with the potential to escape vaccine protection. mRNA vaccines based on the Spike protein have been widely used to prevent COVID-19 illness and have been shown to elicit a protective immune response against VOCs after multiple doses^5,6^. Both SARS-CoV-2 and the mRNA vaccines rely on viral RNA polymerases (RNAPs) for their respective replication or synthesis. RNAPs misincorporate nucleotides at much higher frequencies than their DNA counterparts^3^, generating RNA variants during synthesis which can fuel viral evolution. The mutation frequency of RNA viruses ranges from 10^−4^ to 10^−6^ per base^7^. SARS-CoV-2 is commonly believed to acquire mutations more slowly than other RNA viruses due to the proofreading activity of its exonuclease during RNA synthesis^8^. However, to this point, no empirical studies have directly measured the frequency of RdRp errors during replication (Extended Data Fig. 1a), which is a key parameter for modeling virus evolution. Similarly, the nature and frequency of RNA variants generated during vaccine synthesis remains unknown (Extended Data Fig. 1b).

Technical artifacts introduced during library preparation and next generation sequencing (NGS) pose a major challenge to RNA variant detection. To remove these artifacts, new methods that combine RNA-seq with aspects of circle sequencing and molecular barcoding were developed (ARC-seq) to enable accurate variant calling^9,10^. While these advances in consensus sequencing have reduced technical noise, they typically require considerable substrate (≥1 μg of RNA) and are not feasible for low input samples^11^. Another major constraint is that variant discovery is directly correlated with sequencing depth, and depth can be difficult to achieve for rare transcripts or organisms with large and complex genomes. To overcome these limitations, we developed targeted Accurate RNA Consensus sequencing (tARC-seq) (Fig. 1). tARC-seq combines the basic features of ARC-seq with hybrid capture technology for target enrichment to enable deep variant interrogation of low input SARS-CoV-2 samples.

## tARC-seq validation in *E. coli*

We first validated tARC-seq in *Escherichia coli*, where hybrid capture produced a >30-fold enrichment on average in unique consensus reads across a panel of twelve genes (Extended Data Fig. 2). This enrichment allows us to make high confidence measurements of variant frequencies by gene, depending on baseline expression levels.

## RNA variants in WT SARS-CoV-2

We next used tARC-seq to examine SARS-CoV-2 RNA isolated from infected Vero cells. Since the yield is low, *E. coli* mRNA was added during library preparation to serve as a carrier. With hybrid capture some carrier RNA and host sequences are found in the final library; these can be analyzed separately and serve as internal technical controls. When aligned to the *E. coli* genome, these off-target reads recapitulate the known variant frequency for bulk *E. coli* mRNA (Fig. 2a)^12^.

Using tARC-seq, we achieved on average >16,000X depth in *consensus* reads across the 29,903-nucleotide genome of WT SARS-CoV-2. Positions were filtered for ≥50X depth, which excluded only 0.1% of the genome. Clonal and subclonal variants present at >5% allele frequency were discounted to enrich for *de novo* events (Extended Data Fig. 3a, Supplementary Table 1). We determined that three or more cDNA copies (i.e. minimum family size) was sufficient to filter out most technical artifacts during consensus calling without compromising read depth (Extended Data Fig. 3b). The overall RNA variant frequency in WT virus was 1.16 × 10^−4^, or approximately one in 10,000 nucleotides, meaning new virions harbor on average three novel mutations each (Fig. 2b). These *de novo* variants arise from RdRp errors during genome replication. Host RNA editing by enzymes like APOBECs may also contribute, as evidenced by the elevated frequency of C>T transitions (Fig. 2c and Extended Data Fig. 4)^13^. Expectedly, most variants are base substitutions (8.76E-5), followed by deletions (2.49E-5) and insertions (3.22E-6). Nearly 70% of point mutations were nonsynonymous (Fig. 2d). Classic mutagenesis studies have previously shown that most novel mutations are deleterious or neutral^14,15^, which may account for the modest number of viral variants that have emerged during the pandemic in spite of the high mutation frequency we observe^16^.

To assess whether RNA variants are randomly distributed across the SARS-CoV-2 genome, frequencies were calculated by position. We found that variant frequencies are highly variable across positions and RNA features (Fig. 2f, Extended Data Fig. 5). Our analysis identified 779 hot spots (Extended Data Fig. 5d), or positions with elevated variant frequencies, and 272 cold spots in WT virus (Supplementary Table 2). Moreover, frequencies differed significantly among the open reading frames (ORFs) (Fig. 2e, Extended Data Fig. 6). For example, ORFs encoding structural proteins like Spike are more susceptible to RNA variants, while regions encoding enzymes appear more resistant. These results suggest that some genomic regions in SARS-CoV-2 may mutate faster or could be under higher selective pressure. To find a molecular basis for this variability, nucleotide identity was analyzed across all hot and cold spots and we observed a strong GC bias at positions with significantly elevated variant frequencies (Fig. 2h).

## RNA variants in Alpha and Delta

As SARS-CoV-2 has evolved into several different lineages characterized by specific mutations and VOCs, we next examined whether variant frequencies differ between viral lineages.

Applying tARC-seq to the B.1.1.7 isolate (Alpha), we measured an RNA variant frequency of 1.51E-4 (Fig. 2b). Significantly more point mutations were observed in Alpha, particularly G>A substitutions, which occurred twice as often in Alpha as in WT (Fig. 2c). For DNA-dependent RNAPs (DdRp), these G>A errors are a signature of reduced proofreading^9^. We speculate that the increase in G>A events may reflect less RdRp proofreading in the Alpha lineage. While there are a few candidate mutations in Nsp12 and other replication/transcription complex interactors (Supplementary Table 1), additional studies are needed.

Position-wise calculations in Alpha again revealed a number of hot and cold spots for RNA variants (Extended Data Fig. 5d, Supplementary Table 3), of which 30.6% were shared with WT virus (Supplementary Table 4). Analyzing variant frequencies by ORFs and genomic features, there was 66.7% concordance with WT results (Extended Data Fig. 6). Genes encoding critical proteins such as Spike and RdRp were identified in both samples as having elevated and reduced variant frequencies, respectively (Fig. 2e).

The B.1.617.2 isolate (Delta) had a similar RNA variant frequency (1.43E-4) as Alpha (Fig. 2b). Despite being cultured to the same titer as previous isolates (10^5^ to 10^6^ pfu/mL), tARC-seq produced fewer consensus reads for Delta. While read depth was sufficient for genome-wide studies, positional variant frequencies are not reported as the tests were underpowered.

Altogether, ultra-deep sequencing across three different lineages has revealed a relatively high error frequency for SARS-CoV-2’s RdRp, despite having proofreading activity.

## Viral RdRp prone to template switching

During negative strand synthesis in coronaviruses, RdRp jumps from transcription-regulatory sequences located upstream of most gene bodies (TRS-B) to a leader sequence (TRS-L) in the 5’ UTR to generate subgenomic mRNAs^17^. This programmed template switching is driven by sequence complementarity between TRS sequences, and it functions to add a common leader sequence to viral transcripts to enhance gene expression^18^. tARC-seq detected fusion transcripts in WT SARS-CoV-2 with junctions mapping to canonical TRS sites (Fig. 3a). Moreover, programmed template switching impacts RdRp fidelity as TRS flanking regions exhibit significantly higher variant frequencies in WT and Alpha virus (Fig. 3b, Extended Data Fig. 7c–d).

Non-programmed template switching has also been implicated in insertion events and the emergence of novel coronavirus strains^19^. Analyzing tARC-seq data, we observe many recurrent junctions outside canonical TRSs in fusion transcripts (Extended Data 7a–b) as previously reported^17^. Compellingly, a number of large insertions and deletions were observed in WT virus (Fig. 3d) and related lineages (Extended Data Fig. 8a), many of which appear templated from within the SARS-CoV-2 genome. We model two deletions by RdRp slippage at neighboring repeat sequences during transcription (Fig. 3e). Also shown is a large 41-nucleotide insertion (Fig. 3f), which can be modeled by microhomology-mediated template switching involving three sequential jumps between discrete genomic loci. These templated indels represent the rare scenario where RdRp realigns to the correct sequence after a template switching event. Sequence complementarity between donor and acceptor sites facilitates the jump; however, it is unclear what other features are involved in promoting template switching. As further evidence, we found that many indels are clustered around certain sequences, which we’ve termed transcription “skip sites” (Fig. 3c, Extended Data 8c, Supplementary Tables 5-6). Jumpy RdRp activity at skip sites fuels a diverse repertoire of indels detectable by tARC-seq at a single locus (Extended Data Fig. 8b). For example, the indel frequency at position 23308, pictured in Fig. 3e–f, is elevated ten-fold over the genome-wide average in both WT (g.23303: 2.97E-4) and Alpha (g.23308: 3.11E-4). Skip sites often sit adjacent to regions of microhomology (Extended Data 8d) and homopolymeric nucleotide runs (Extended Data 8e), which drive up local indel frequencies.

## Non-programmed template switching drives pandemic genomic epidemiology

Signatures of aberrant RdRp template switching are present in sequences from real-world pandemic data as well (Fig. 4a). One event, a GGG to AAC substitution, defines the 20B clade (Fig. 4b) from which the Alpha, Gamma, Lambda and Omicron lineages evolved. All of these viral variants also contain lineage-specific multiple nucleotide alterations that can be modeled as single RdRp misalignment and realignment events templated from within the SARS-CoV-2 genome (Fig. 4c–e; Extended Data Fig. 9). Thus, template switching represents a major driver of virus evolution.

## RNA variants in the Pfizer-BioNTech COVID-19 vaccine

**Extended Data Figure 1 |:**
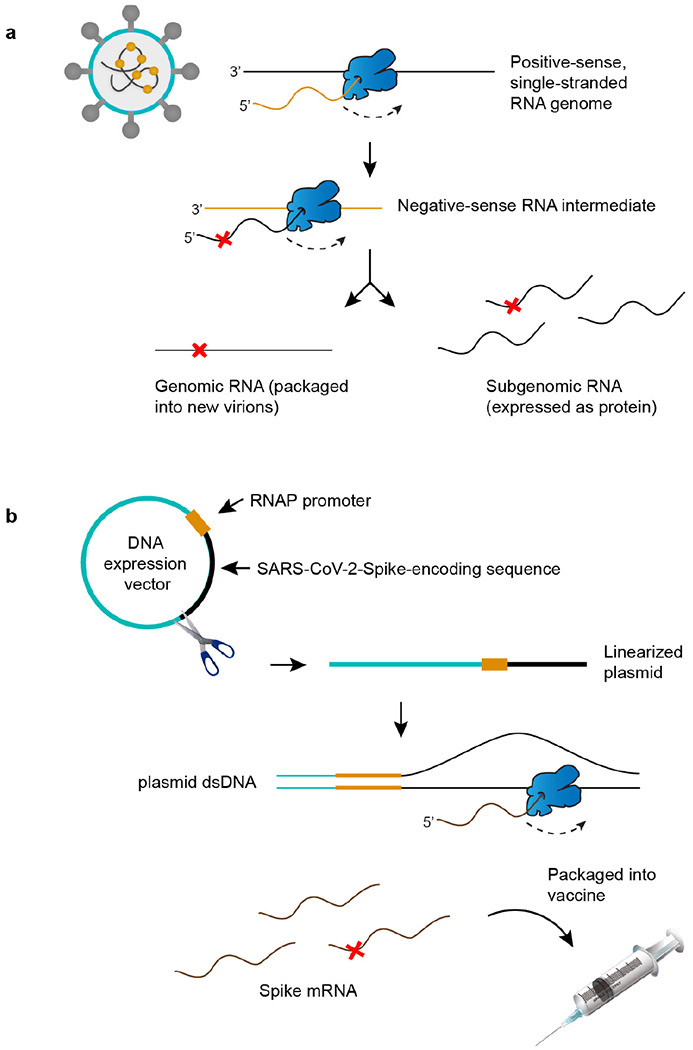
Origins of RNA variants in SARS-CoV-2 virus and its mRNA vaccines. **a**, As a positive-strand RNA virus, SARS-CoV-2 encodes an RNA polymerase (RNAP, in blue) that is responsible for both replication and gene expression. RNAP errors (red ‘X’) generate genetic diversity in SARS-CoV-2 and fuel the evolution of novel strains. **b,** Pfizer-BioNTech COVID-19 vaccine production begins with a SARS-CoV-2-Spike-encoding sequence that is GC-enriched and codon-optimized. The template is placed downstream of the T7 promoter in a plasmid expression vector and linearized for *in vitro* transcription (IVT). T7 RNAP errors during IVT generate sequence diversity in mRNA vaccines. Needle syringe art attributed to DataBase Center for Life Science^41^.

**Figure 1 |:**
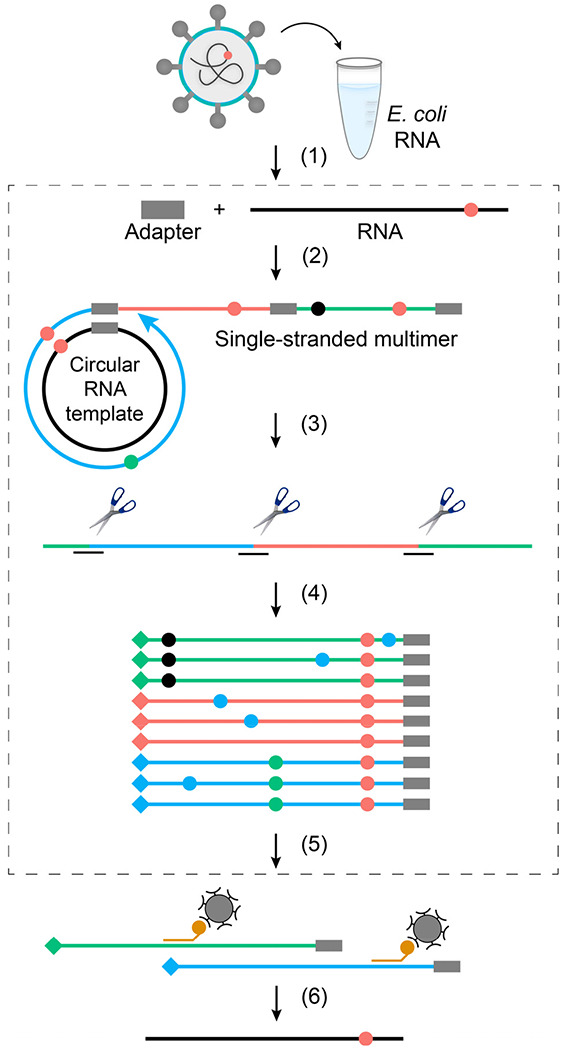
Library preparation for tARC-seq. (1) SARS-CoV-2 RNA is added to a carrier and the sample is fragmented. (2) Fragments are ligated to barcoded adapters, circularized and primed for rolling-circle reverse-transcription. (3) The resulting cDNA multimers are restriction digested into monomer copies. (4) Sequencing adapters and additional barcodes (♦♦♦) are added through subsequent PCR steps. (5) SARS-CoV-2 reads are enriched through hybrid capture, followed by post-capture PCR. (6) Final library is sequenced, reads are organized into families by barcode, and collapsed into consensus sequences. This process of error correction removes technical artifacts (●●●) and identifies true RNA variants (●) that occur at the same position across duplicates. The non-targeted sister protocol to tARC-seq is outlined in grey (steps 2-4, 6).

**Figure 2 |:**
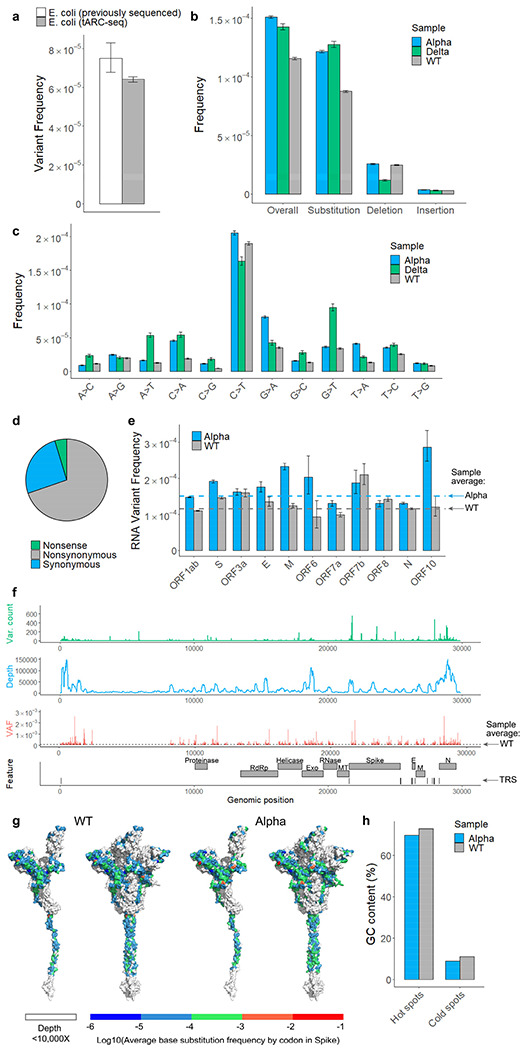
Spectrum and frequency of RNA variants in SARS-CoV-2. **a**, tARC-seq reproduces known variant frequencies in *E. coli.* **b**, RNA variants were measured in ancestral SARS-CoV-2 (WT), the B.1.1.7 lineage (Alpha), and the B.1.617.2 lineage (Delta) using tARC-seq. Variants occurred at a frequency of 1.16 x 10^−4^ in WT virus, with higher rates observed in both Alpha and Delta. **c**, RNA variants were dominated by C>T and G>A transitions. **d**, Most variants are nonsynonymous. **e**, Genes encoding structural proteins like Spike show higher variant frequencies (Fisher exact test). **f**, Mapping variant allele fractions (VAF) by position across the SARS-CoV-2 genome reveals an uneven landscape. **g**, Base substitution frequencies by codon mapped against Spike protein illustrate the distribution of hot and cold spots for RNA variants. **h**, RNA variant hot spots show strong GC bias *in vivo*. Error bars represent Wilson score 95% confidence intervals. For analysis, a maximum 5% clonality cutoff was applied to the data and positions were filtered for ≥50X depth. A more stringent depth filter (≥10,000X) was applied to the position-wise analyses **(f, g)** to minimize skewing due to inadequate sampling.

**Figure 3 |:**
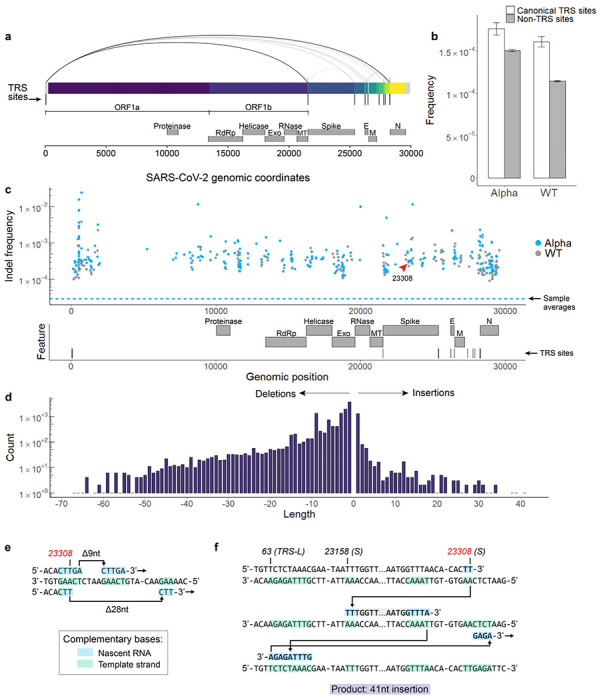
RdRp template switching at sites of sequence complementarity models rare events in SARS-CoV-2. **a**, Using a spliced aligner (STAR) for mapping, chimeric reads are detected in WT virus. Recurrent jumps between canonical TRSs are visualized as arcs connecting the 5’ and 3’ ends of each chimeric junction. These jumps signify programmed RdRp template switching that functions in viral gene expression. **b**, TRS regions had a higher RNA variant frequency compared to control regions in both WT and Alpha, suggesting that programmed polymerase jumping reduces overall fidelity in these regions. **c**, Among other low-fidelity regions are indel hot spots, or loci with significantly elevated frequencies of insertions and deletions. Indel hot spots are calculated by Fisher exact test, filtered for ≥10,000X depth, and graphed by position for both Alpha and WT. **d**, The size spectrum of insertions and deletions in WT virus reveals rare, large events, many of which appear templated from within the SARS-CoV-2 genome. **e**, **f**, Templated indels can be explained by non-programmed RdRp jumping and realignment at sites of sequence complementarity outside of canonical TRSs. Three events from tARC-seq data are modeled, all occurring at the same indel hot spot in the S gene (g.23308, indicated by the red arrow in panel **c**). The full sequence of the 41-nt insertion (**f**) is: TGGTTAAAAACAAATGTGTCAATTTCAACTTCAATGGTTTA.

**Figure 4 |:**
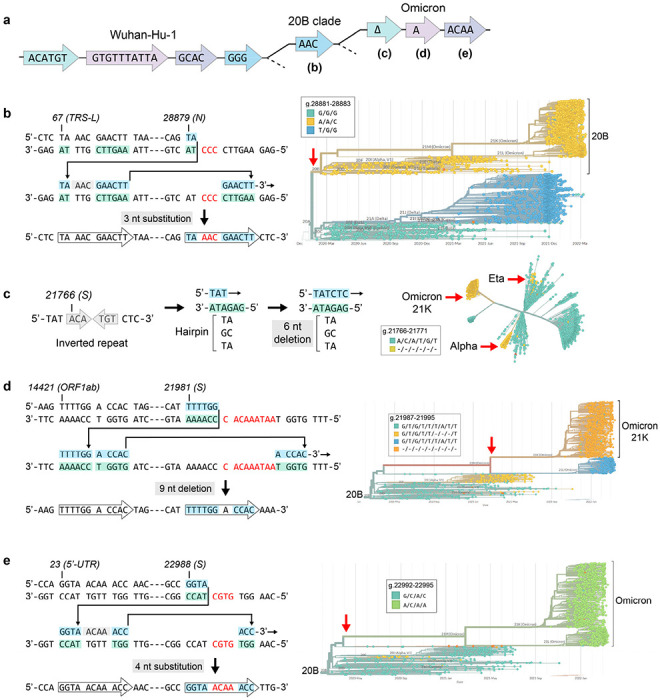
On the origin of Omicron. Pandemic data shows that many complex mutations in SARS-CoV-2 appeared suddenly (red arrows). They likely did not accumulate gradually but were driven by a single event: RdRp template switching. In the events modeled here, 3’ complementarity facilitates the misalignment and realignment of RdRp, creating complex mutations that have fueled viral evolution. **a**, Phylogenetic tree based on sequence alterations that define the 20B and Omicron clades; not drawn to any scale. Discrete, coordinated nucleotide alterations are coded by color, and each template switching event is mapped out below (**b-e**). **b**, A GGG>AAC mutation in the N gene occurred once early in the pandemic and helped define the 20B clade. **c**, A small hairpin in S has spawned the same 6-nt deletion on more than 3 separate occasions, while other single events are specific to Omicron (**d-e)**. Phylogenetic trees were constructed in Nextstrain v2.35.0^32^ from genomes sequenced between Dec. 2019 and March 2022.

The Pfizer-BioNTech COVID-19 vaccine was the first mRNA vaccine approved for use and has been an instrumental tool in our public health arsenal against SARS-CoV-2^20^. Vaccine mRNA is the product of *in vitro* transcription (IVT) by T7 phage polymerase from a codon-optimized, Spike-encoding DNA construct (Extended Data Fig. 1b)^21^. As a DdRp, T7 polymerase also commits errors during transcription at frequencies ranging from 10^−4^ to 10^−6[22,23]^. These errors have not been studied in the context of vaccine production or vaccine-induced immunity. As vaccine mRNA is abundant and amenable to sequencing by bulk RNA consensus sequencing (ARC-seq) (Fig. 1), we characterized the frequency and spectrum of RNA variants in the Pfizer vaccine.

**Figure 5 |:**
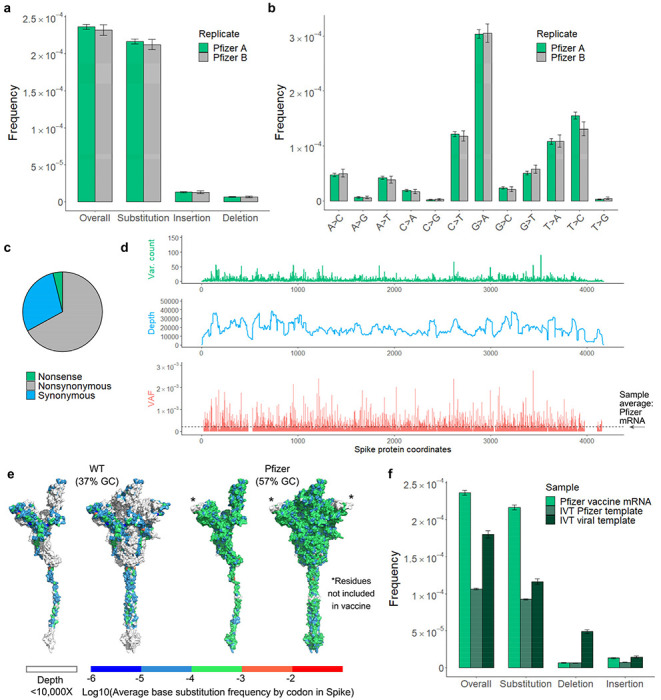
Spectrum and frequency of RNA variants in the Pfizer-BioNTech COVID-19 mRNA vaccine. Total ARC-seq was applied to SARS-CoV-2 Spike mRNA purified from the Pfizer-BioNTech COVID-19 vaccine to assess the fidelity of T7 RNA polymerase in vaccine production. **a**, The variant frequency for vaccine mRNA is 2.24 x 10^−4^ (n=2). Samples come from separate vials (labeled Pfizer “A” and “B”) of the same lot. G>A transition was the dominant event subtype in vaccine samples (**b**) and most variants were nonsynonymous (**c**). Overall, the type of variants observed in Spike differs between vaccine and viral samples. **d**, **e,** Positional frequencies are less variable and fewer hot spots are observed in the vaccine (VAF = variant allele fraction). **f**, Compared to T7 IVT transcripts, vaccine mRNA has significantly more variants, and high GC content was associated with fewer polymerase errors *in vitro.* Analysis as in Figure 2.

The overall variant frequency in vaccine mRNA is 2.34 x 10^−4^, or double that of WT SARS-CoV-2 (Fig. 5a). At that frequency, full-length Spike transcripts contain on average one novel variant each. The spectrum and clonality of variants in the S gene detected by (t)ARC-seq differs between vaccine and WT virus (Fig.5, Extended Data Fig. 3b), likely reflecting differences between the two RNAPs. Fewer deletions, but more insertions, were observed in vaccine mRNA (Fig. 5a). The C>T base substitution frequency is lower in the vaccine given the absence of RNA editing *in vitro* (Fig. 5b). Additionally, G>A transitions were more frequent compared to any of the viral isolates tested (Fig. 5b), which may reflect the intrinsic lack of proofreading activity of T7 RNAP^22^. Among the point mutations, 67% were nonsynonymous (Fig. 5c). Sequencing depth was more consistent across the S gene in vaccine mRNA and position-wise tests revealed a more even landscape of variant frequencies compared to viral samples (Fig. 5d–e).

## RNA errors during T7 *in vitro* transcription

The high variant frequency in the Pfizer vaccine could stem from a number of variables, including template codon optimization, T7 polymerase biology, synthesis with modified nucleotides, IVT conditions, and vaccine packaging and storage. To address this, a series of T7 IVT reactions was performed in parallel over a range of temperatures on two different templates: (1) the native S gene from WT SARS-CoV-2, and (2) the codon-optimized Spike construct from the Pfizer vaccine. The two templates differ significantly by nucleotide content, with the modified vaccine template being 57% GC compared to 37% for the viral template. mRNA from either IVT reaction had a reduced variant frequency compared to the vaccine (Fig. 5f), but differences between the IVT reactions were apparent as well (Extended Data Fig. 10a–b). Significantly, IVT from the vaccine template produced fewer RNA variants of all types (1.06E-4) at an overall rate comparable to WT virus, suggesting that high GC content is protective against transcription errors *in vitro.* We observed that IVT reactions from the viral template are seven times more prone to insertion errors (4.9E-5 for insertions; 1.8E-4 overall) compared to the vaccine template, implicating low GC content in template switching (Extended Data Fig. 10a). We also found evidence of template switching in IVT transcripts (Extended Data Fig. 10d), corroborating our findings in viral isolates. Modulating the temperature during IVT did not appear to affect the variant frequency (Extended Data Fig. 10a–b).

After controlling for GC enrichment and standard T7 IVT conditions, we conclude that the intrinsic error-prone nature of T7 RNAP is the main source of vaccine variants. However, other features specific to Pfizer vaccine production, such as the use of N1-methylpseudouridine^24,25^, mRNA purification, liposomal packaging, vaccine freezing and storage, or the scale up for production, also contribute to the RNA variant frequencies observed in the vaccine samples..

## Discussion

Herein, we describe a targeted sequencing method for detecting RNA variants in rare transcripts and low abundance samples. We have sequenced three SARS-CoV-2 isolates and established a baseline variant frequency of ~1 in 10,000 per nucleotide for the virus. While higher than other predictions^4^, this frequency is comparable to similar observations in poliovirus, which also utilizes an RdRp for replication but lacks an associated proofreading activity^8,11,26^. The error frequency estimations were previously based on the presence of a proofreading 3’-to-5’ exoribonuclease (ExoN, nsp14) in SARS-CoV-2 that is distinct from the viral RdRp^27^. The same proofreading activity has been implicated in promoting template switching^28^, which we show here is error-prone. Thus, our work highlights the promiscuous nature of SARS-CoV-2’s RdRp driven by nucleotide misincorporation and erroneous template switching, both controlled by the same exonuclease. ExoN may be a key protein involved in tuning viral evolution. Together, these results showcase the fundamental biology propelling viral diversity and evolution on a massive scale during the COVID-19 pandemic.

In conjunction with viral data, we also measured RNA variants in the Pfizer BioNTech COVID-19 vaccine using ARC-seq. At a frequency of 1 in 5,000 nucleotides, the pace of vaccine variants appears balanced against viral evolution and suggests that the majority of mRNA produced encodes variant Spike proteins. The role of vaccine heterogenicity in the immune response is currently unknown. Our data may provide insight to explain how mRNA vaccines against COVID-19 offer broader protection against novel strains upon boosting^22,29–31^. Vaccine variants could promote a more diverse immune repertoire, which offers benefits in the context of a rapidly evolving virus. However, other uses like cancer vaccines or mRNA drugs may require high fidelity transcription to reduce the risk of autoimmunity or improve clinical efficacy^2^. Identification and usage of high fidelity RNAP may be crucial for the development of future therapies. Importantly, our results build on a growing body of work promoting mRNA-based drug technologies in medicine and public health.

Finally, beyond the COVID-19 pandemic, tARC-seq has revealed many new principles concerning basic RdRp biology. RNA errors are non-random and linked to GC content, transcriptional patterns and sequence complementarity. RdRp is capable of non-programmed template switching to form structural variants, insertions and deletions, ultimately fueling virus evolution. Future research should expound on the sequence motifs and rules that regulate RNA errors and polymerase switching events as they likely afflict every corner of life. Perhaps then we can begin to understand how organisms have evolved to mitigate RNAP infidelity or learned to exploit it.

## Methods

### RNA extraction

All RNA samples were processed under RNase-free conditions using dedicated equipment and reagents. To maintain RNA integrity, samples were limited to ≤1 freeze-thaw cycle, kept on ice whenever possible, and not subjected to high temperatures (≥65 C) in the presence of metal cations ^9^. RNA integrity was confirmed via Agilent Tapestation™ prior to sequencing library preparation.

#### SARS-CoV-2

Ancestral SARS-CoV-2 was received from the World Reference Center for Emergeing Viruses and Arboviruses at The University of Texas Medical Branch (Galveston, TX, USA) under the direction of Drs. Scott Weaver and Kenneth Plante. Alpha variant (SARS-CoV-2, hu/USA/CA_CDC_5574/2020, Source: Centers for Disease Control and Prevention, BEI Catalogue number NR-54011) and Delta variant (Isolate hCoV-19/USA/PHC658/2021 (Lineage B.1.617.2; Delta Variant, Source: St. Jude’s Children’s Research Hospital, BEI Catalogue Number NR-55611) were received from BEI Resources. Viral stocks were prepared by infecting Vero CCL-81 cells as previously described^33^. Briefly, 48-72 hours post infection, supernatant from infected cultures were collected and a plaque assay was performed using Vero E6 cells to calculate viral titer. Virus was inactivated in TRIzol™ reagent before freezing at −80 C for long-term storage. RNA was exacted from thawed TRIzol preps ^34^ at the time of library preparation.

#### Pfizer-BioNTech COVID-19 vaccine

Vaccine was collected from the remains of unexpired, spent vials (LOT ER8727, EXP 07/2021) that had been stored at room temperature for <6 hours, as per the manufacturer’s recommendations for proper handling. No vaccine that could otherwise have been administered to patients was sacrificed for the purposes of this study. Vaccine samples were transferred on wet ice. Upon receipt, RNA was immediately extracted with PhenokChloroformTsoamyl Alcohol (25:24:1) (PCA) followed by ethanol precipitation. Samples were reconstituted in nuclease-free water and analyzed by Qubit Fluorometer™ and Agilent Tapestation™ prior to storage at −80 C.

#### Escherichia coli

Luria broth was inoculated from isolated colonies and grown for 16 hours at 37 C. The next day, overnight cultures were washed, diluted 100-fold in fresh LB, and grown at 37 to mid-log phase (OD600 ~0.4). 1 mL culture aliquots were then harvested in duplicate and placed on ice preceding RNA extraction using the RNAsnap™ protocol^35^. Following harvest, sample cleanup was performed with the RNA Clean & Concentrator Kit (Zymo Research) and DNase treatment (TURBO™ DNase) was applied off-column at 37 C for 1 hour. The ribosomal RNA fraction was depleted via the RiboMinus™ Transcriptome Isolation Kit for bacteria (Invitrogen™), and the resulting mRNA was concentrated by ethanol precipitation for downstream library preparation.

#### *In vitro* transcription

Two different templates were used for *in vitro* transcription (IVT): (I) the native SARS-CoV-2 Spike gene (Addgene 145670), and (II) the codon-optimized, GC-enriched spike-encoding construct from Pfizer-BioNTech ^36,37^. Both genes were expressed from bacterial expression plasmids downstream of T7 promoters. Plasmids were isolated from *E. coli* cultures using the QIAprep Spin Miniprep Kit™ and subjected to single-enzyme digest with SnaBI (viral template) or Mlul (vaccine template). Linearized plasmid was then cleaned up by PCA and verified by Qubit Fluorometer™ and gel electrophoresis before IVT. Reactions were performed in parallel at escalating temperatures (30, 37, 42 C) using the Hi Scribe® T7 High Yield RNA Synthesis Kit (NEB) and the ProFlex PCR System Thermal Cycler (Applied Biosystems). Starting from 200 ng of template, reactions were allowed to proceed for two hours before TURBO™ DNase treatment. Transcription products were purified by PCA for sequencing. Notably, while IVT from the vaccine template was less efficient and had lower yields, vaccine transcripts were more resistant to RNaseIII fragmentation during library preparation.

### Library preparation and sequencing

#### Total accurate RNA consensus sequencing (ARC-seq)

1 μg of RNA was enzymatically fragmented with RNaseIII for 7 min to an average size of 450 nt ^38^. Following PCA cleanup, the remaining steps of library construction were guided by ARC-seq design ^10^. Briefly, RNA was 5’ adenylated and ligated to unique molecular identifiers (UMIs). Barcoding individual fragments *before* any amplification reactions downstream is critical for error correction during consensus calling. The library was then circularized and primed for rolling-circle reverse-transcription, yielding multiple conjoined copies of the original fragment. After digesting these long cDNA oligomers, the individual monomers were PCR amplified with tailed primers to add sequencing adapters and additional indexes. The final library was analyzed by Tapestation prior to paired-end sequencing on the Illumina NextSeq 550 system. Importantly, reaction conditions were optimized throughout to reduce RNA damage from heat and metal ion catalysis.

#### Targeted accurate RNA Consensus sequencing (tARC-seq)

For concentrated samples (≥ 100 ng/μL), fragmentation can proceed as described above. However, for low-abundance samples it is recommended to add carrier RNA up to 1 μg. For the SARS-CoV-2 experiments, a previously sequenced *E. coli* sample was mixed 4:1 with viral RNA and served as both the carrier and internal control. Once fragmented, the targeted libraries are prepared by the total RNA protocol up through the last indexing step. SARS-CoV-2 reads were then enriched using the COVID xGen Hybrid Capture Kit (IDT) and 7-9 cycles of post-capture PCR to generate the final library.

### Data analysis

#### Error-correction and variant calling

Illumina BCL files were converted to Fastq and demultiplexed from the 6 nt sample barcode in the i7 index read (base masking: Y*,I6N*,Y*,Y*). Next, UMIs were extracted and appended to the read headers before converting to unaligned BAM format. Specifically, the 11 nt PCR UMI was read from the i5 index, and the 16 nt cDNA UMI was clipped from the leading 16 bases of Read 2. Reads with identical UMI tags, originating from the same RNA fragment, were grouped into families and collapsed into consensus reads using a custom python script. Consensus FASTQ sequences were aligned to the appropriate reference genome using BWA-MEM (accessions: NC_000913.3, NC_045512.2). Reads were then quality filtered, clipped, and realigned with GATK v3.8. Finally, variants were called using mpileup and tabulated via a custom R script. (Scheme S1)

#### Statistical information

Frequencies were calculated as variant count / total consensus nucleotides sequenced. Proportion confidence intervals (two-tailed) were calculated for each frequency and error bars represent Wilson scores of 95% confidence. To detect positions in the SARS-CoV-2 genome that have a base substitution frequency different from the sample average, we constructed a contingency table and performed Fisher’s exact test comparing each position to the genome-wide average. All positions that passed our initial filter (clonality ≤0.05, raw depth ≥50, fraction of N ≤0.05) were included in this analysis. To control the false discovery rate, p-values were adjusted using the Benjamini–Hochberg procedure. Positions with adjusted p-values <0.05 and substitution frequencies that are elevated or reduced compared to the genome-wide average were called as hot and cold spots, respectively. *E. coli* hybrid capture was performed on 2 biological replicates. Viral analysis was performed on 1 biological replicate per genotype. Vaccine sequencing data represents 2 vials of the same lot (n=2). *In vitro* experiments were performed in triplicate at 30, 37, and 42 C (n=1 per temperature).

#### 3D protein map of variant frequencies by codon

Average RNA base substitution frequencies were calculated for each codon in the Spike protein by sum(base substitution count)/sum(adjusted depth). All positions that passed our initial filter (clonality ≤0.05, raw depth ≥50, fraction of N ≤0.05) and were sequenced at a raw depth ≥10000 were included in this analysis. The Spike protein full-length model 6vsb_1_1_1 ^39^ was then colored based on the codon average RNA base substitution frequency using Pymol ^40^.

## Extended Data

**Extended Data Figure 2 |:**
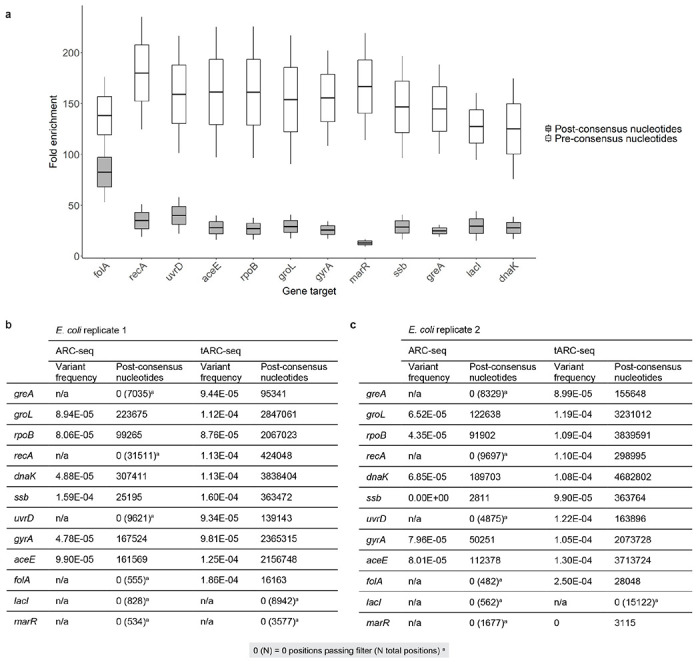
Validation of tARC-seq in *E. coli.* **a,** tARC-seq achieves >30-fold enrichment in post-consensus nucleotides across a 12-gene panel in E. coli (n=2). PCR duplicates account for most of the pre-consensus nucleotides sequenced, and fold-enrichment drops during consensus calling as duplicates of the same parent RNA fragment are collapsed into a single read. The drop in enrichment between pre- and post-consensus reads is more pronounced for low-expression genes like marR. This reflects both the efficacy of probe binding and the scarcity of marR transcripts. Fold enrichment was calculated from the cumulative, normalized sequencing depth across each gene in tARC-seq samples versus matched bulk ARC-seq controls. **b-c,** RNA variant frequency analysis by gene is poorly powered using the original ARC-seq method^10^. Coverage is highly variable between targets leading to inaccurate estimates of the true variant frequency. Combining ARC-seq with hybrid capture (tARC-seq) significantly enriches for reads across a 12-gene panel in *E. coli,* increasing the statistical power of the study (n=2, reported separately).

**Extended Data Figure 3 |:**
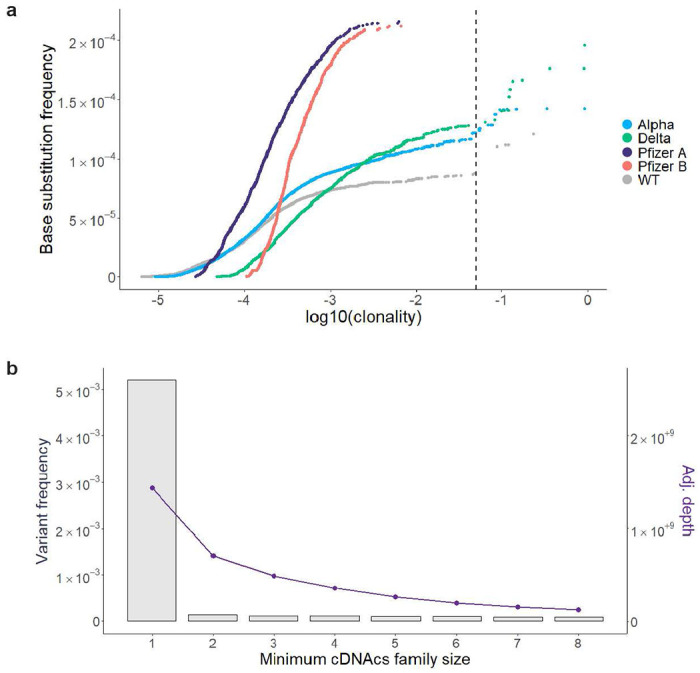
Empirical validation of tARC-seq data analysis parameters. **a,** In contrast to *de novo* variants, clonal and subclonal variants are not independent events and should be filtered out during analysis. They typically arise from a single RdRp error and are subsequently propagated through viral replication, inflating the true error frequency. To determine an appropriate cutoff, all variants were graphed by the cumulative base substitution frequency as a function of each variant’s clonality. Relatively few clonal outliers were discovered in the Pfizer vaccine compared to viral samples. A cutoff of 0.05 – or ≤5% allele fraction – counted most variants on the curve while excluding clonal outliers. **b,** The overall variant frequency (left y-axis, grey bars) in WT SARS-CoV-2 is graphed by consensus read depth (right y-axis, purple line) over a series of minimum cDNAcs family sizes (minmem2). Minmem2 is an expression of the minimum number of PCR copies required to form a cDNA consensus sequence during consensus calling. A family size of 1 is equivalent to traditional RNA-seq without error correction, while a family size of 3 was previously found to sufficiently correct for technical artifacts^11^.

**Extended Data Figure 4 |:**
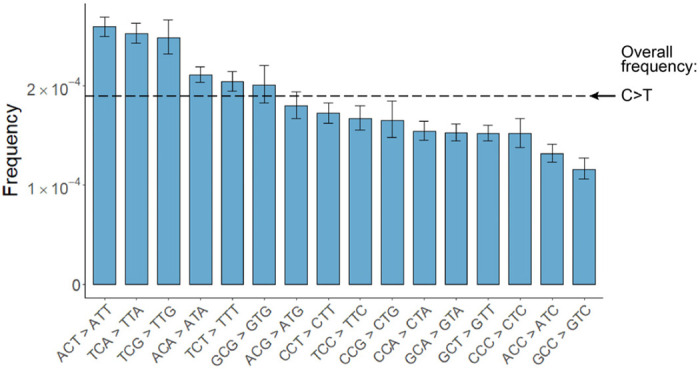
Variant frequencies at RNA editing motifs. Cytosine residues across the SARS-CoV-2 genome have high rates of C>T variants. Frequencies are further elevated at RNA editing motifs, suggesting that APOBEC and ADAR activity may mutagenize viral transcripts and genomes *in vivo*^13^.

**Extended Data Figure 5 |:**
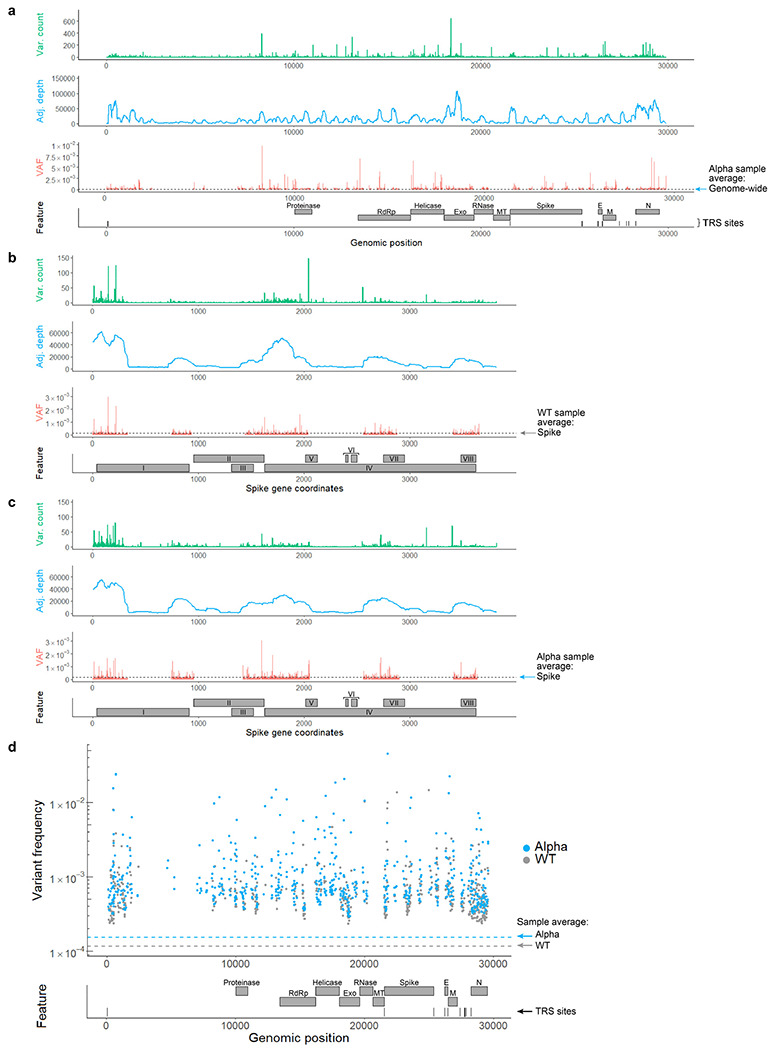
RNA variant frequencies by position in WT and Alpha SARS-CoV-2. **a,** A genome-wide map of variant counts, sequencing depth and variant frequencies by position in the Alpha isolate (VAF = variant allele fraction). **b-c,** Mapping positional base substitution frequencies across the S gene. Feature legend: (I) N-terminal domain, (II) Receptor-binding domain, (III) Receptor-binding motif, (IV) Subdomains 1-2, (V) S1/S2 cleavage region, (VI) Fusion peptides, (VII) Heptad repeat 1, (VIII) Heptad repeat 2. Most variants cluster in the N-terminal domain, downstream of the TRS site for Spike. **d,** Hot spots for RNA variants in both WT and Alpha are mapped by genomic position and feature. These loci had significantly elevated variant frequencies compared to the genome-wide average (p-value <0.05 by Fisher’s exact test with Benjamini-Hochberg correction). Positions were filtered for depth ≥ 10,000 to reduce skew in low coverage regions.

**Extended Data Figure 6 |:**
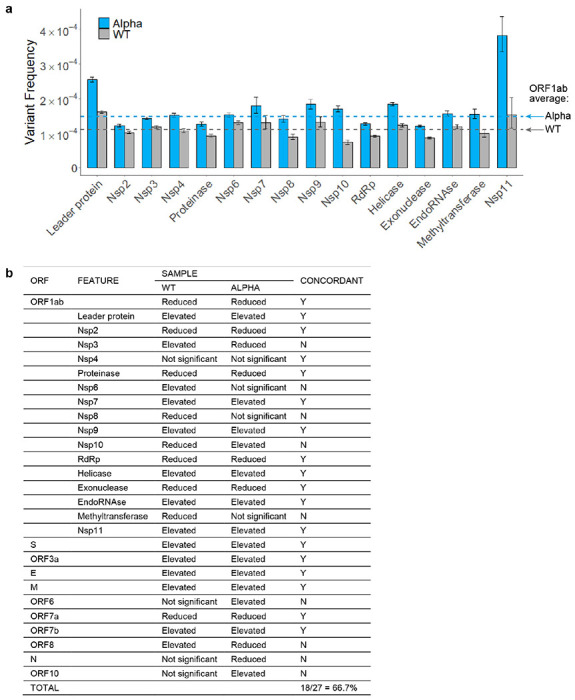
RNA variant frequencies by feature in WT and Alpha SARS-CoV-2. **a,** Among nonstructural features in ORFlab, variant frequencies were significantly elevated in the leader protein. However, regions encoding critical enzymes like RdRp, exonuclease, and proteinase were relatively protected. Error bars represent 95% Wilson confidence intervals. **b,** The various ORFs and features were previously graphed by variant frequency (panel **a** above, and Fig. 2e). Functional regions with significantly elevated or reduced frequencies relative to the sample average are indicated here (p-value <0.05 by Fisher’s exact test with Benjamini–Hochberg correction). Column 5 indicates whether the results from each region were concordant between Alpha and WT, with overall agreement exceeding 66%. As with the calculations for hot and cold spots, differences in sequencing depth between samples, particularly for smaller regions, can impact the results.

**Extended Data Figure 7 |:**
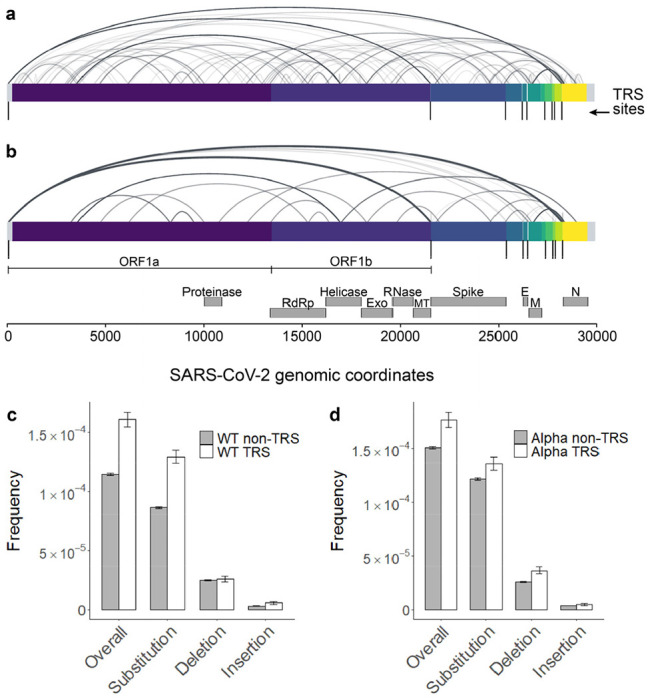
Interaction between transcription regulatory sequences and RdRp fidelity. tARC-seq detected chimeric junctions between canonical TRSs in WT SARS-CoV-2 (Fig. 3a). **a,** However, many of the observed junctions lay outside canonical regions, suggesting non-programmed template switching by a promiscuous polymerase. **b,** While tARC-seq has the sensitivity to detect single events, the data was filtered further to include only high confidence junctions with ≥100 observations. Even after increasing the stringency to remove potential ligation artifacts (Fig. 1, step 2), many non-canonical junctions remained. Each arc represents a chimeric alignment where the left and right x-intercepts correspond to the 5’ and 3’ junction coordinates and line shading reflects frequency. **c-d,** While TRS regions comprise only ~3.5% of the SARS-CoV-2 genome, they incur RNA variants at a higher frequency in both WT and Alpha virus. Each TRS region (n=10) is small (<115 nt) and composed of one canonical TRS site plus 100 flanking nucleotides.

**Extended Data Figure 8 |:**
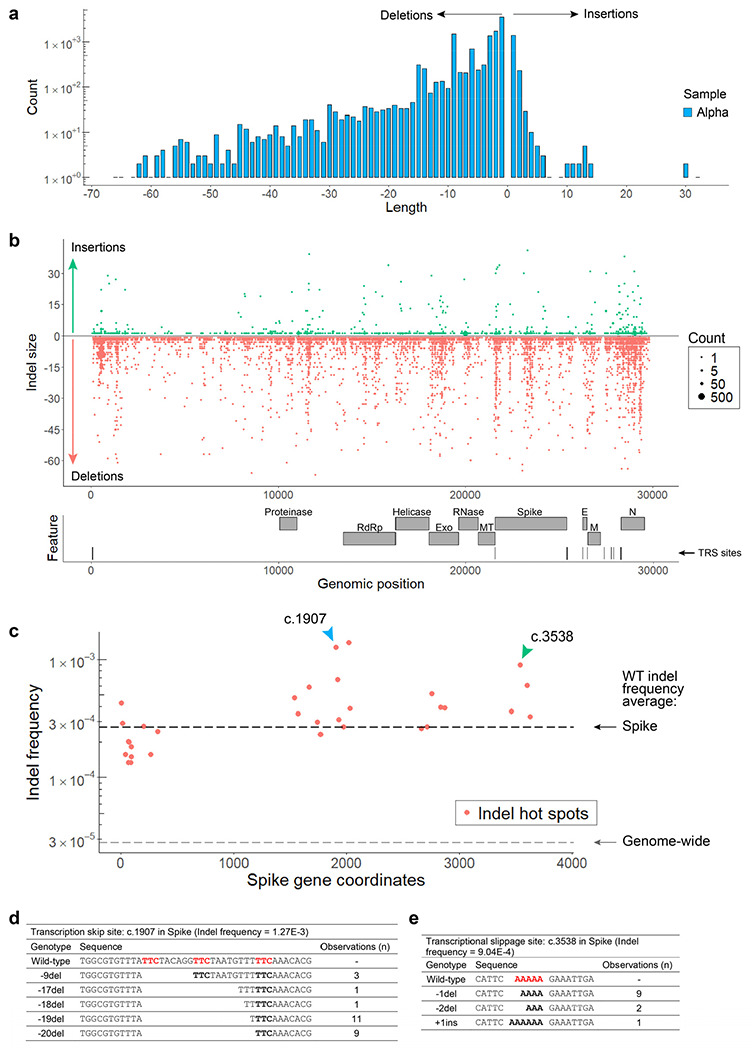
Characterizing indel hot spots in SARS-CoV-2. **a,** In Alpha, single nucleotide insertions and deletions predominate with additional peaks around multiples of 3 that preserve the reading frame, as expected. Many large indels suggestive of RdRp template switching were also observed. **b,** Indels are mapped by size (y-axis) and count (dot size) across the SARS-CoV-2 genome. Promiscuous RdRp activity at skip sites fuels a diverse repertoire of indels detectable by tARC-seq at a single locus. **c,** Indel hot spots observed in WT virus across the S gene are graphed by coordinate and frequency. Overall, Spike had higher rates of indels compared to the genome-wide average. **d-e,** The spectrum of indels at two particular hot spots (c.1907 and c.3538, indicated by colored arrows) are reviewed in detail. **d,** With adjacent regions of microhomology (red) to drive local template switching, an array of large deletions was discovered at c.1907. This position was also identified as an indel hot spot in the Alpha variant and many of the same deletions (−9del, −18del, −19del, −20del) were observed in that sample. This pattern is suggestive of a transcription skip site where the sequence and local genome architecture promote RNAP template switching and high variant frequencies. Interestingly, none of the large deletions we found at c.1907 in Spike, including the in-frame events, are seen in pandemic data, suggesting they are deleterious. In support of this, Nextstrain data shows this region to be highly conserved across SARS-CoV-2 lineages and other members of the Coronaviridae family. **e,** Homopolymeric nucleotide runs (red) can also trigger indel hot spots through transcriptional slippage events, as shown at c.3858 in Spike. Hot spots represent positions with significantly elevated indel frequencies as determined by Fisher’s exact test (p-value <0.05 with Benjamini–Hochberg correction).

**Extended Data Figure 9 |:**
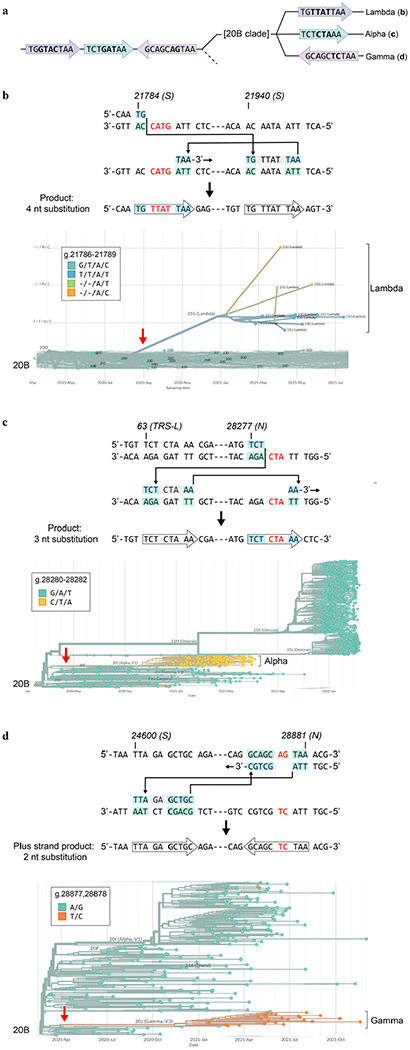
RdRp template switching drives genomic epidemiology during the COVID-19 pandemic. Variant-specific multiple nucleotide alterations can be modeled as singular RdRp template switching events based on 3’ micro-complementarity that facilitates RdRp misalignments/realignments. **a**, Phylogenetic tree based on sequence alterations observed in variants arising from the 20B clade; not drawn to any scale. The different colors indicate variant-specific nucleotide alterations. **b-d**, Top panels, proposed template switching models that explain multiple nucleotide alterations in the Lambda, Alpha, and Gamma lineages. Bottom panels, phylogenetic trees that establish the singular origin of the coordinated multiple nucleotide alterations in each lineage. Phylogenetic trees were constructed in Nextstrain v2.35.0^32^ from genomes sequenced between Dec. 2019 and March 2022.

**Extended Data Figure 10 |:**
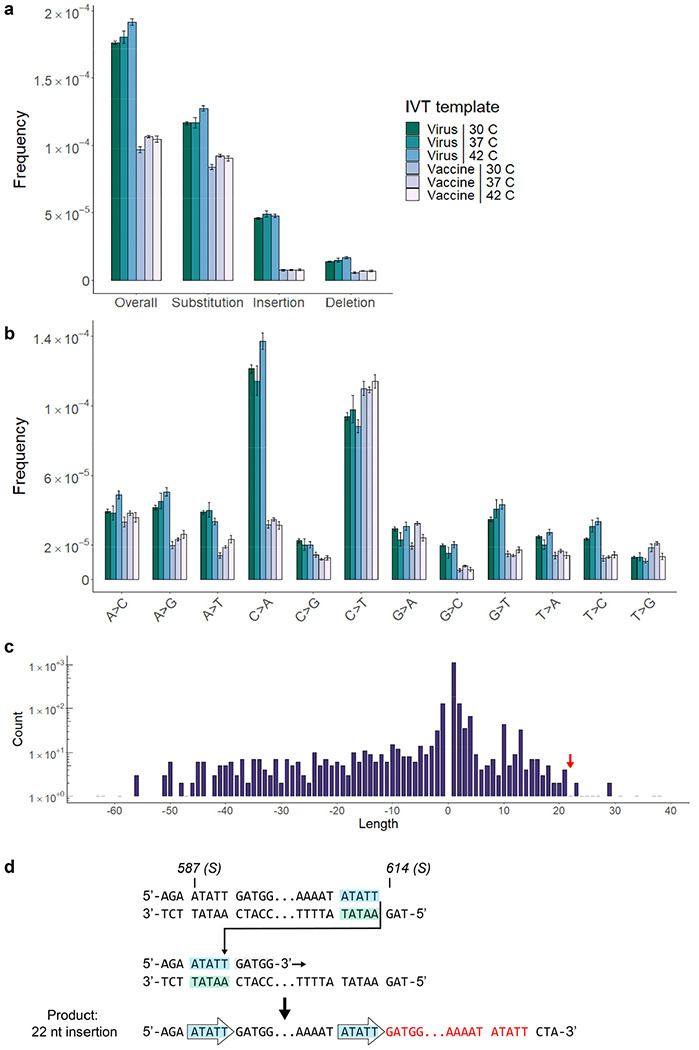
RNA variants across T7 in vitro transcription reaction conditions. **a-b,** Spectrum of RNA variants from a series of T7 *in vitro* transcription reactions. Two templates are compared in parallel: the S gene from WT SARS-CoV-2 (virus), and the GC-enriched Spike-encoding sequence from Pfizer (vaccine). Reactions were performed using a commercial T7 RNAP kit over a range of active temperatures (30, 37, 42 C). **c,** A broad distribution of indels is seen in IVT transcripts from the viral Spike template at 37 C. As with RdRp *in vivo*, many large events appear to be mediated by RNAP template switching. **d,** A 22 nt insertion observed in IVT transcripts (red arrow, panel **c**) is modeled by microhomology-mediated template switching. (Insertion sequence: ATATTGATGGTTATTTTAAAAT).

## Figures and Tables

**Table 1 |:** Glossary of key terms.

WT virus	Ancestral SARS-CoV-2 (Isolate USA-WA1/2020)
Viral variant or lineage	Virus that harbors fixed, characteristic genomic mutations compared to WT (e.g. Alpha, Delta, Omicron)
RNA variant	Any base alteration from the WT genomic RNA sequence (e.g. G>A)
Clonal variants	Fixed RNA alterations present at a given loci across sequenced observations (clonality > .9)
Subclonal variants	RNA alterations present in <90% of consensus sequences but >5%
De novo variants	A new RNA variant that arises from RNAP errors, RNA modification or damage and not from propagation of an existing variant during replication (clonality < 0.05)

## Data Availability

Sequencing data are available through the Sequence Read Archive under Bio Project PRJNA824595. All other data are available from the corresponding author upon reasonable request.
